# Application of Long-Range Surface Plasmon Resonance for ABO Blood Typing

**DOI:** 10.1155/2016/1432781

**Published:** 2016-12-22

**Authors:** Wanida Tangkawsakul, Toemsak Srikhirin, Kazunari Shinbo, Keizo Kato, Futao Kaneko, Akira Baba

**Affiliations:** ^1^Graduate School of Science and Technology and Center for Transdisciplinary Research, Niigata University, 8050 Ikarashi 2-Nocho, Nishi-ku, Niigata 950-2181, Japan; ^2^Materials Science and Engineering Programme, Multidisciplinary Unit and Center of Intelligence Materials and Systems, NANOTEC Center of Excellence at Mahidol University, Department of Physics, Faculty of Science, Mahidol University, Rama 6 Rd., Phayathai, Rajathavee, Bangkok 10400, Thailand

## Abstract

In this study, we demonstrate a long-range surface plasmon resonance (LR-SPR) biosensor for the detection of whole cell by captured antigens A and B on the surface of red blood cells (RBCs) as a model. The LR-SPR sensor chip consists of high-refractive index glass, a Cytop film layer, and a thin gold (Au) film, which makes the evanescent field intensity and the penetration depth longer than conventional SPR. Therefore, the LR-SPR biosensor has improved capability for detecting large analytes, such as RBCs. The antibodies specific to blood group A and group B (Anti-A and Anti-B) are covalently immobilized on a grafting self-assembled monolayer (SAM)/Au surface on the biosensor. For blood typing, RBC samples can be detected by the LR-SPR biosensor through a change in the refractive index. We determined that the results of blood typing using the LR-SPR biosensor are consistent with the results obtained from the agglutination test. We obtained the lowest detection limits of 1.58 × 10^5^ cells/ml for RBC-A and 3.83 × 10^5^ cells/ml for RBC-B, indicating that the LR-SPR chip has a higher sensitivity than conventional SPR biosensors (3.3 × 10^8^ cells/ml). The surface of the biosensor can be efficiently regenerated using 20 mM NaOH. In summary, as the LR-SPR technique is sensitive and has a simple experimental setup, it can easily be applied for ABO blood group typing.

## 1. Introduction

Blood group typing is necessary in the treatment of patients with massive blood loss. Matching the donor's blood group with the patient's blood group is required before transfusion to avoid a blood incompatibility event. The ABO blood typing system is tested first for all blood transfusions, because it can cause serious damage in all other blood systems due to its strong specific antigen-antibody interactions. The ABO blood group system is classified based on the inherited properties of red blood cells (RBCs). It is determined by the presence or absence of certain proteins and oligosaccharides called antigens, including A and B, which are presented on the surface of RBCs. A, B, and O RBCs structures are similar, but the difference is that A-type RBCs have N-acetylgalactosamine (GalNac) and B-type RBCs have galactose (Gal), while O-type RBCs have neither. The blood group is associated with Anti-A and Anti-B immunoglobulin M antibodies (IgM), which are the body's natural defense against foreign antigens. The ABO blood typing system breaks blood types down into four groups: A, B, AB, or O. Each group specifies the antigens and antibodies found in that individual, such as the A antigens and B antibodies found in blood group A, the B antigens and A antibodies found in blood group B, both A and B antigens found in blood group AB, and both A and B antibodies found in blood group O [1–5].

Surface plasmon resonance (SPR) technique has become a widely used technique for the detection of biomolecular interactions since the 1990s. Among other applications, SPR sensor is often used in clinical diagnosis for the detection of antigen-antibody binding, protein-ligand interaction, and DNA detection [6–12]. SPR is a sensitive technique that can be applied for real time monitoring of the biomolecular interactions in solution [13, 14]. The SPR biosensor is based on changes of the optical reflectivity by the adsorption of biomolecules on a gold (Au) surface, which cause a change in refractive index near the SPR-active gold surface. Long-range surface plasmon resonance (LR-SPR) involves surface plasmons (SPs) that propagate along a thin metallic film embedded between two dielectric materials with similar refractive indices, for which both the evanescent field intensity and the penetration depth (more than 1 *μ*m) are greater than those for conventional short-range SPs (penetration depth is ~200 nm) [15–20]. Hence, it is expected that the sensitivity at longer distances from the metal surface will increase with LR-SPR spectroscopy. Recently, Homola et al. reported that the LR-SPR biosensor could detect large analytes, such as the bacteria* Escherichia coli* (0.7–1.0 *μ*m) due to the large penetration depth of the evanescent wave [21, 22]. In addition, the LR-SPs enhanced the optical field wave at the metal-dielectric interface, leading to a higher sensor sensitivity and increased penetration into the analyte solution than that observed for conventional SPR. As a result, a thicker sensor coating with a significantly larger number of binding analyte molecules on the surface could be used [23, 24]. We have also previously reported an LR-SPR immunosensor based on the electrospun poly (acrylic acid) (PAA) fibers for the detection of human IgG [20].

The detection of antigens A and B on the surface of RBCs by conventional SPR was reported by Quinn et al. [25, 26]. However, the detection of RBCs via conventional SPR is limited due to the penetration depth of evanescence field (~200 nm), which is much less than the size of RBCs (i.e., ~2 *μ*m thick and ~7.5 *μ*m in diameter). Recently, various studies have been reported in the applications of SPR technique for the study of RBCs. Houngkamhang et al. [27] reported ABO blood typing via SPR imaging which observed interaction between immobilized Anti-A and Anti-B antibodies array and A and B antigens on RBCs surface. The interaction of antigens around lower RBCs membrane surface and immobilized Anti-A and Anti-B antibodies array was applied to obtain the agglutination strength of the RBCs and immobilized antibody [28]. The SPR imaging, which utilized the shear force generated within the flow cell, was used to measure the rolling speed of red blood cell [28]. The technique can only extract the information at the interface because the limit of the evanescence field makes it impossible to study in whole cell. A number of important pieces of information regarding the property of the cell were undetectable such as cell elasticity and cell deformation. Krupin et al. used long-range surface plasmon waveguides for capturing only blood group antigen A on RBCs by immobilized Anti-A IgG via no comparative studies with Anti-B [29]. To our knowledge, there was no report regarding the detection of RBC by LR-SPR. Because the difference in surface chemistry, experimental set-up, and nature of SPR signal between LR-SPR and SPR, in this work, it is desirable to explore the possibility of applying LR-SPR to detect ABO blood group and understand the nature of the LR-SPR signal. Anti-A and Anti-B will be covalently immobilized on self-assembled monolayer (SAM) surface and A, B, AB, and O will be detected by using LR-SPR. The Cytop (*n* = 1.34) fluoropolymer is used in the LR-SPR system in order to match the refractive index with the phosphate buffered saline (PBS) buffer. All the results of blood typing were consistent with the results obtained from the agglutination test. The lowest detection amounts of RBC-A and RBC-B were 1.58 × 10^5^ and 3.83 × 10^5^ cells/ml indicating that LR-SPR has much higher sensitivity than that obtained with conventional SPR biosensors [25, 26] and LR-SPR waveguides [29]. We demonstrate that LR-SPR is a good candidate for classification of blood typing and has a high potential in other clinical applications such as bacteria cells, cancer, and rare RBCs.

## 2. Experimental

### 2.1. Chemicals and Materials

The 11-mercaptoundecanoic acid (11-MUA), phosphate buffered saline (PBS) tablets, and sodium acetate buffer (pH 5) were all purchased from Sigma-Aldrich. The 1-ethyl-3-(3-dimethylaminopropyl)-carbodiimide hydrochloride (EDC), N-hydroxysuccinimide (NHS), and ethanolamine hydrochloride (EA-HCl) were purchased from Tokyo Chemical Industry (TCI). The CTL-809M and CTL-180 solvents for the Cytop solution were purchased from Asahi glass. Mixed clones of monoclonal Anti-A and Anti-B and standard A cells, B cells, and O cells were obtained from the research unit of the Thai Red Cross Society.

### 2.2. LR-SPR Instruments and LR-SPR Sensor Chip Fabrication

**Figure 1:**
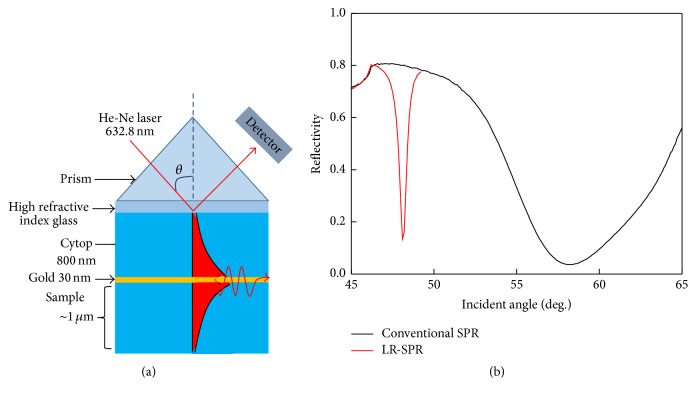
(a) Schematic of the long-range surface plasmon resonance (LR-SPR) setup. (b) The angular reflectivity curves of conventional SPR (prism-glass slide/gold (50 nm)/PBS buffer) and LR-SPR (prism-glass slide/Cytop (800 nm)/gold (30 nm)/PBS buffer).

The Kretschmann configuration is used for exciting surface plasmons using He-Ne laser with wavelength (*λ*) of 632.8 nm [30, 31]. LR-SPR was arranged to propagate along a thin metallic film embedded between two dielectrics with similar refractive index (Figure 1(a)). For the fabrication of the LR-SPR sensor chip, a 7% Cytop solution (dissolved 9% Cytop (CTL-809M) in CTL-180 solvent) was spin-coated on a high-refractive index glass (OHARA S-LAH60, *n* = 1.83) at a first spin rate of 500 rpm for 10 s and second spin rate of 1300 rpm for 20 s. The Cytop solvent was dried at 180°C for 1 h in an oven, and the Cytop film (ca. 800 nm) with a refractive index of 1.34 (similar to a refractive index of water, 1.33) was obtained on a high-refractive index glass. Then, 30 nm of gold (Au) film was deposited on the Cytop film by vacuum evaporation. A chromium layer (1 nm) was used to promote Au adhesion with the Cytop film. As shown in Figure 1(b), the angular reflectivity curves of the conventional SPR and LR-SPR chips measured in a bare Au/PBS buffer system indicate that the curve of the LR-SPR chip is sharper than that of the conventional SPR chip.

### 2.3. Immobilization of Antibodies and Detection of RBCs

**Figure 2:**
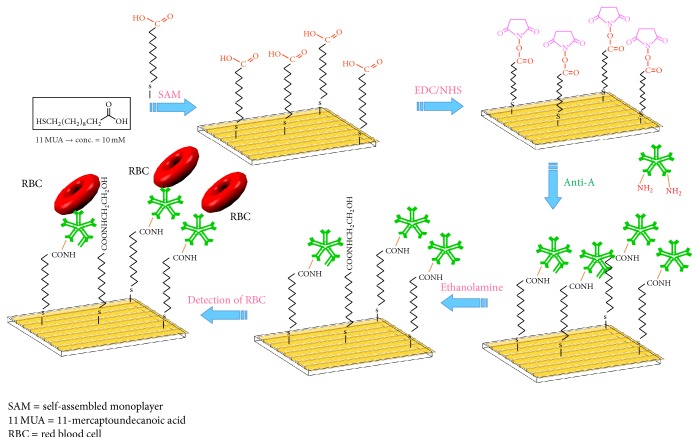
Schematic representation of immobilization of antibodies (Anti-A/Anti-B) and detection of red blood cells (RBCs).

The LR-SPR chip Au surface was covered by a self-assembled monolayer (SAM) using 10 mM of 11-mercapto-undecanoic acid (11-MUA) in ethanol. The carboxylic groups of the SAM surface were activated to their ester forms by immersion in 0.4 M EDC and 0.1 M NHS dissolved in deionized water at a ratio of 1 : 1. The monoclonal antibody for blood group A (Anti-A) and monoclonal antibody for blood group B (Anti-B) were covalently immobilized on the activated sensor surface (after rinsing the sensor chip with PBS buffer) by injecting the antibodies in sodium acetate at pH 5 at a 1 : 10 dilution onto the activated SAM surface [27]. The residual activated surface sites, which did not react with antibodies, were inactivated or blocked with 0.2 M ethanolamine. The RBC samples from standard A, B, and O cells were detected on the immobilized antibody by observing the change in the refractive index on the LR-SPR biosensor. Standard RBC-A and RBC-B samples were diluted in the range of 3 × 10^4^ to 3.8 × 10^7^ cells/ml. The number of cell number was counted using the hematocrit test. Regeneration of the LR-SPR chip surface was performed using 20 mM NaOH, followed by rinsing with PBS running buffer. The LR-SPR measurements were carried out in a nonflow condition. A summary of the antibody immobilization on the biosensor chip and the detection of RBCs is shown in Figure 2.

## 3. Results and Discussion

### 3.1. Immobilization of Antibody and Detection of RBC-A

**Figure 3:**
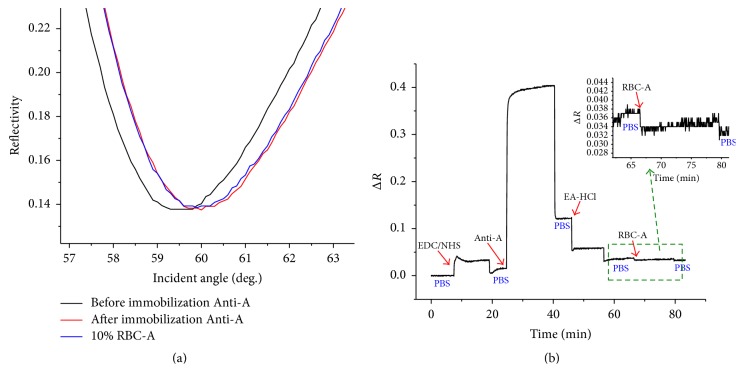
(a) Angular reflectivity curves of conventional SPR before and after immobilization and after the detection of RBC-A. (b) Corresponding SPR kinetic reflectivity curve.

We compared the sensing ability between conventional SPR and LR-SPR for the detection and classification of RBC typing on grafted SAMs (11-MUA). First, the conventional SPR sensor chip was used for the evaluation of the immobilization of Anti-A/Anti-B on the sensor chip and the detection of RBC-A. Angular reflectivity curves of the conventional SPR were observed before immobilization or after grafting the SAM, after immobilization of Anti-A, and after the detection of RBC-A (Figure 3(a)). The corresponding kinetic reflectivity curve is also shown in Figure 3(b). The angular SPR reflectivity curve after immobilization of Anti-A was shifted to higher angle, indicating that Anti-A was immobilized on the sensor chip. The increase in the reflectivity during the immobilization is also shown in the SPR kinetic curve (Figure 3(b)). However, the reflectivity during the detection of RBC-A is almost constant, as shown in the kinetic curve (Figure 3(b)). Moreover, the corresponding angular reflectivity curve was almost constant after the detection of RBC-A. This indicates that the conventional SPR sensor is limited to the detection of RBC, presumably because of the fact that the evanescence field of conventional SPR is much shorter than the thickness of RBCs, and hence the reflectivity change is not sensitive to the adsorption of the surface.

**Figure 4:**
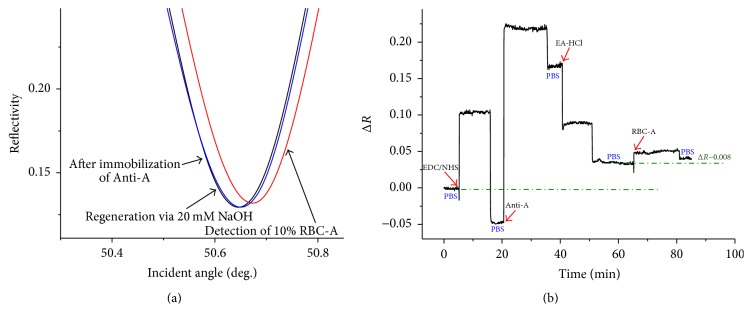
(a) Angular reflectivity curves of LR-SPR after immobilization of Anti-A, after detection of RBC-A, and after regeneration with 20 mM NaOH. (b) Corresponding LR-SPR kinetic reflectivity curve.

Then, the LR-SPR sensor chip was used for the detection and classification of RBC typing on 11-MUA SAM. The LR-SPR kinetic reflectivity curve for the immobilization of Anti-A and the detection of RBC-A is shown in Figure 4(b). The Anti-A was immobilized on activated group on the SAM surface. The remaining available active groups were blocked by ethanolamine. The baseline in the figure is obtained after the activation process by rinsing with PBS buffer. The reflectivity was obviously increased by the immobilization of Anti-A and after the detection of RBC-A (by ~0.008) (Figure 4(b)). In this result, the baseline of the initial reflectivity is relatively high because the experiment was continuously carried out after the activation of the surface. Hence, the reflectivity was saturated by the injection of Anti-A. However, after the detection of RBC-A, we clearly observed the increased reflectivity change compared to the conventional SPR. In order to clearly compare with the conventional SPR reflectivity curve, the *y*-scale was shown by finite difference of reflectivity, Δ*R*. Furthermore, the angular SPR reflectivity curve clearly shows that the dip angle shifts to a higher angle after the detection of RBC-A (Figure 4(a)). These results indicate that the LR-SPR is more sensitive for the detection of RBC-A in comparison with the conventional short-range SPR measurement.

### 3.2. Surface Regeneration

**Figure 5:**
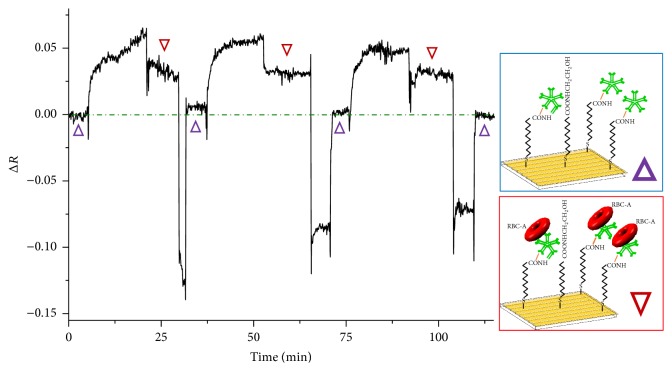
LR-SPR kinetic curve showing detection of red blood cells (RBCs) and regeneration of the surface using 20 mM NaOH. PBS buffer was used to obtain a baseline reading (Δ) and then RBC-A is bound on the Anti-A for ~5 min, before the surface is rinsed with PBS buffer (∇). Then, 20 mM NaOH is injected on the RBC-A/Anti-A surface, followed by the injection of PBS buffer (Δ).

Because one important advantage of the biosensor is reusability, we also studied the regeneration ability of RBC-A on Anti-A. For the regeneration of the RBC detection system, we found that 20 mM of NaOH was a suitable condition to disrupt the antigen on the RBCs, causing the disruption of the RBC-antibody specific adsorption interaction without destroying immobilized antibody on the activated surface. The LR-SPR curve is shifted back to the lower incident angle and is almost the same as the LR-SPR curve of immobilized Anti-A surface, indicating that the adsorbed RBC-A is completely removed from the Anti-A surface without removing the immobilized Anti-A (Figure 4(a)).  Figure 5 shows the kinetic property of the LR-SPR during the regeneration of RBC-A/Anti-A surface over three times. During the experiment, PBS buffer was used to obtain a baseline reading, which is shown as “Δ” in the figure. After the binding of RBC-A on the Anti-A for about 15 min, the surface with residual unbound RBC-A was rinsed with PBS buffer, which is shown as “∇.” Then, 20 mM NaOH was injected on the RBC-A/Anti-A surface, followed by the injection of PBS buffer (baseline, shown as “Δ”). As shown in the figure, each time the baseline returned to the original baseline values, indicating that the Anti-A surface still remained (i.e., was regenerated), even after three sensing and regeneration processes.

### 3.3. Immobilization of Antibody and Detection of RBC-B

**Figure 6:**
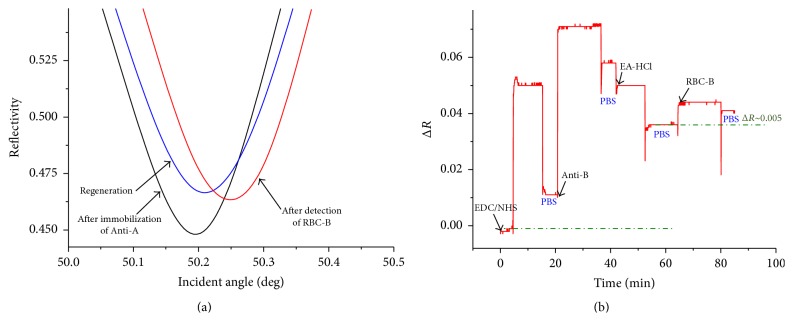
Angular reflectivity curves for LR-SPR after immobilization of Anti-B, after detection of RBC-B, and after regeneration with 20 mM NaOH. (b) Corresponding LR-SPR kinetic reflectivity curve.

Next, we immobilized Anti-B on activated 11-MUA SAM surface and detected RBC-B by LR-SPR (Figure 6). In this experiment, the sensing procedure was the same as for RBC-A detection described above. Similar to the results for RBC-A, LR-SPR showed an obvious increase at each adsorption step, indicating that it can easily detect RBC-B.

### 3.4. Specificity between Antibodies and RBCs

**Figure 7:**
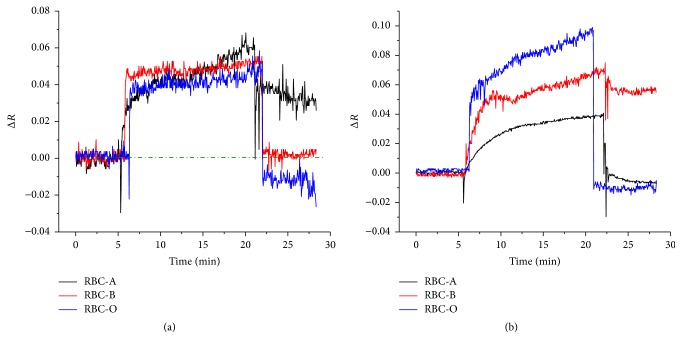
LR-SPR kinetic curves during the detection of RBC-A, RBC-B, and RBC-O on Anti-A (a) and on Anti-B (b).

To study the specificity between antibodies and RBCs, Anti-A or Anti-B was immobilized on activated 11-MUA SAM surface, and RBC-A, RBC-B, or RBC-O was detected to determine both specific and nonspecific interactions. The LR-SPR kinetic curves during the detection of RBC-A, RBC-B, and RBC-O on Anti-A (Figure 7(a)) and Anti-B (Figure 7(b)) show that the reflectivity was increased in all cases. This indicated that the RBCs were adsorbed on the antibodies both by specific and nonspecific interactions. After rinsing with PBS buffer, the LR-SPR reflectivity for RBC-B and RBC-O decreased to around baseline. This is because the nonspecifically or physically adsorbed RBCs were removed from the Anti-A surface. On the other hand, the LR-SPR reflectivity for RBC-A kept higher reflectivity than that of the initial baseline, indicating that the specifically adsorbed RBC-A on Anti-A remained on the surface. The slight decrease by the PBS buffer indicates some physically adsorbed RBC-A was removed from the surface. In the case of the RBCs detection on Anti-B surface, the LR-SPR reflectivity for RBC-A and RBC-O decreased to almost the initial baseline after the adsorption on the surface, while the reflectivity for RBC-B remained higher. These results indicate that the specific and nonspecific adsorption of RBCs can be clearly detected by the LR-SPR sensor chip, showing the ability to classify blood types.

### 3.5. Determination of the Detection Limit

**Figure 8:**
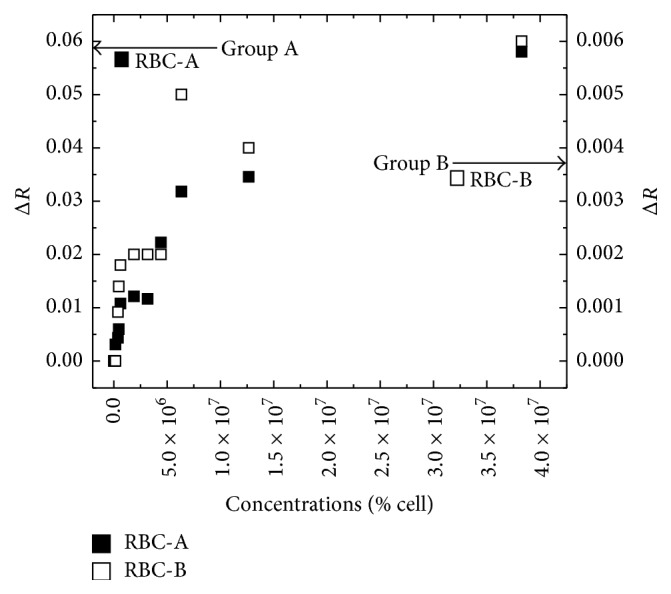
LR-SPR reflectivity change after sensing with various concentrations of red blood cells (RBCs).

Standard RBC-A and RBC-B samples were serially diluted from their original concentration and injected to study the interaction with Anti-A and Anti-B immobilized on 11-MUA SAM. RBC-A and RBC-B were detected on the immobilized Anti-A and Anti-B, respectively, by varying each concentration. RBCs at each concentration were detected for 10 min, followed by rinsing with PBS. The SPR reflectivity change (Δ*R*) was obtained by the reflectivity difference between the baseline before the injection of RBCs and after rinsing with the PBS buffer. Antibody surfaces were regenerated with 20 mM NaOH for the following experiment. As shown in Figure 8, the reflectivity change (Δ*R*) of LR-SPR increases when the concentration of RBC-A and RBC-B increases. The limit of detection (LOD) of RBC-A and RBC-B were at 1.58 × 10^5^ cells/ml and 3.83 × 10^5^ cells/ml, respectively. The LOD was defined as three times standard deviation of the blank (PBS buffer) [32]. We found that the obtained LOD using the LR-SPR is lower than that with conventional short-range SPR (i.e., 3.3 × 10^8^ cells/ml) [25, 26] and also lower than that with long-range surface plasmon waveguides that exhibited the LOD less than 3 × 10^5^ cell/ml [33]. This clearly indicates that LR-SPR is a promising technique for the detection of RBCs.

## 4. Conclusion

We demonstrated that the LR-SPR sensor, consisting of high-refractive index glass, Cytop film layer, and thin gold (Au) film, is capable of detecting large analytes, red blood cell (RBCs). The antibodies of blood group A and group B (Anti-A and Anti-B) are able to be covalently immobilized on a grafting self-assembled monolayer (SAM)/Au surface on the LR-SPR biosensor chip. For blood typing, the RBC samples are detected on the immobilized Anti-A and Anti-B surface by the change in the refractive index. We found that the results of blood typing obtained by the LR-SPR biosensor were consistent with those obtained from the agglutination test. Moreover, the LR-SPR exhibited the lowest detection limits of 1.58 × 10^5^ cells/ml for RBC-A and 3.83 × 10^5^ cells/ml for RBC-B, indicating that the LR-SPR chip has a higher sensitivity than conventional short-range SPR biosensors (3.3 × 10^8^ cells/ml). Finally, the sensor showed a good efficiency of surface regeneration using 20 mM NaOH. Therefore, the LR-SPR technique demonstrates many advantages for the detection of RBCs and could be used to perform ABO blood group typing in the future.
